# Diagnostic value of IgG antibody and stool antigen tests for chronic *Helicobacter pylori* infections in Ibb Governorate, Yemen

**DOI:** 10.1038/s41598-024-58165-w

**Published:** 2024-03-29

**Authors:** Bashir A. Al Ofairi, Marwan K. Saeed, Mohammed Al-Qubaty, Ahmed. M. Abdulkareem, Majed A. Al-Jahrani

**Affiliations:** 1 Section of Microbiology, Departement of Biological Sciences, Faculty of Science, Sana’a University, Sana’a, Yemen; 2 Pathology Department, Faculty of Medicine, Taiz University, Taiz, Yemen; 3 Department of Medical Laboratories, University of Science and Technology, Ibb, Yemen; 4 Departement of Medical Laboratory, Faculty of Medical Sciences, Queen Arwa University, Sana’a, Yemen

**Keywords:** Immunological techniques, Microbiology techniques, Health care

## Abstract

The stool antigen test (SAT) and the serum *Helicobacter pylori* (*H. pylori*) IgG antibody assays exhibit significant utility in the clinical diagnosis of *H. pylori* infection and in distinguishing between acute and chronic infections. The main objective of the current study was to identify the diagnostic value of serum *H. pylori* IgG antibody and SAT in the detection of *H. pylori* infections among chronic *H. pylori*-infected patients residing in Ibb Governorate, Yemen. 200 patients with *H. pylori* infection, confirmed through positive results in the serum immunochromatographic antibody test, were selected for *H. pylori* infection confirmation using serum *H. pylori* IgG antibodies and SAT across diverse hospitals, gastroenterology, and Hepatology clinics in Ibb Governorate. After the selection of patients, blood and stool specimens were obtained from all participants and underwent analysis via the Statistical Package for the Social Sciences (SPSS). The prevalence of *H. pylori* infection demonstrated variability based on the confirmatory tests, with rates of 54% for SAT and 78.5% for serum *H. pylori* IgG antibody, contrasting with a 100% prevalence observed in the screening serum immunochromatographic antibody test. Clinically, the study categorized *H. pylori* infections into four stages, whereby a significant proportion of patients (40.5%) exhibited positivity for both serum *H. pylori* IgG antibody and SAT, indicative of active chronic infections. The majority of positive cases only manifested serum *H. pylori* IgG antibody presence (chronic infections) at 38%, whereas 13.5% exclusively tested positive for SAT, corresponding to acute infections. Moreover, 88% of patients did not have either serum *H. pylori* IgG antibody or SAT (absence of infections) during confirmatory tests. Noteworthy is the study's approach employing multiple tests for *H. pylori* infection detection, focusing predominantly on chronic infections-prevailing types caused by *H. pylori*. The results revealed a significant association between serum levels of *H. pylori* IgG antibody and SAT results with the presence of diverse gastrointestinal symptoms among patients, which increased with long *H. pylori* infection durations.

## Introduction

*Helicobacter pylori* is a Gram-negative bacterium, measuring 2–4 µm in length and 0.5–1 µm in width usually spiral-shaped but the bacterium can appear as a rod, while coccoid shapes appear after prolonged in vitro culture or antibiotic treatment^1,2^.

The incidence of *H. pylori* infection is nearly 50% of the global population, it affected by low hygienic conditions and poor socioeconomic status. In addition, the incidence of *H. pylori* infections in UK and USA were 13.4% and 27.1%, respectively. While in developing countries, the estimated prevalence was 90% and approximately 4.4 billion individuals worldwide were positive for *H. pylori* infections (more than half the world population). There are a wide variation in the prevalence of *H. pylori* infections between different regions in Yemen, which depend on symptomatic patients with chronic dyspepsia the prevalence of *H. pylori* infections were 75–82.2%^3–6^.

Gastritis defines as any inflammation of the gastric mucosa. Indeed, worldwide *H. pylori* infection is the most common cause of chronic gastritis; it is also the most important risk factor for peptic ulcer and its complications as well as for gastric cancer (GC). *H. pylori* causes progressive damage to the gastric mucosa and plays as a causative role in a number of important diseases, including: duodenal ulcer (DU) disease, gastric ulcer (GU) disease, gastric adenocarcinoma and gastric mucosa associated lymphoid tissue lymphoma. It is estimated that *H. pylori* positive patients have 10–20% lifetime risk of developing ulcer disease and 1–2% have risk of developing distal GC^6–10^.

Various diagnostic techniques are employed for the detection of *H. pylori* infections, including both invasive methods (such as endoscopy for obtaining biopsies for histology, rapid urease test, and culture) and non-invasive methods (such as serology, urea breath test, and SAT). Serological diagnosis of *H. pylori* used to identify antibodies against Hp. The enzyme immunoassay (EIA) test has been the most prevalently used. Most commercial EIA tests are based on detecting immunoglobulin G (IgG), with sensitivity and specificity values ranging from 60 to 100%. Additionally, SAT is able to diagnose an ongoing infection, while the serological tests are limited to diagnose a contact with the bacterium, which can be current or lifetime. The SAT has many positive aspects: it is noninvasive, quick, has good sensitivity, specificity and reliability. This test can be used both for diagnosis of the infection and for monitoring therapy effectiveness, already 4 weeks after the end of treatment, its low cost, easy use and the possibility to collect samples have increasingly widespread the use of this method^11–17^. The current study was designed to evaluate of *H. pylori* infections in Ibb governorate, Yemen using mmunochromatographic antibody testing, serum *H. pylori* IgG antibody testing via EIA, and the SAT method and determine the value of both serum IgG *H. pylori* antibody and SAT in detection the chronic *H. pylori* infections.

## Materials and methods

The current research employed a cross-sectional design involving the collection of stool and blood specimens from 200 patients afflicted with *H. pylori* infections who had tested positive for rapid serum antibody of *H. pylori* between October 2019 and November 2020. Diagnosis of patients based on the detection of serum *H. pylori* antibodies via rapid diagnostic testing. Validation of the results was carried out utilizing the serum *H. pylori* IgG antibody (NovaLisa *Helicobacter pylori* IgG ELISA (HEL G0220) with 96 wells for determinations, manufactured by NOVA TEC IMMUNODIAGNOSTICA GMBH in Germany, exhibiting a specificity of 93.0% and sensitivity of 98%) for chronic infections, and the serum SAT (developed by Intech corporation, showcasing a sensitivity of 98.4% and a specificity of 98.8%) for acute infections at various hospitals, hepatology, gastrointestinal clinics, and different medical laboratories in Ibb Governorate, Yemen. All patients with confirmed *H. pylori* infections, irrespective of age or gender, were considered for inclusion in this study. Exclusion criteria comprised malignancies such as ovarian, endometrial, cervical, lung, breast, liver, gastric, pancreatic, and colorectal cancers, individuals with other gastrointestinal infections, and those who provided incomplete responses in the questionnaire.

A structured and reliable questionnaire was designed and distributed among all participants included in the study. This questionnaire encompassed the following sections: the demographic profiles of patients, including age, gender, Khat chewing (*Catha edulis*) smoking, educational background, dietary patterns, weight, height, familial medical history including incidences of *H. pylori* infections and peptic ulcer disease (PUD), duration of *H. pylori* infection, gastrointestinal symptoms including (nausea, vomiting, heartburn, and abdominal pain, along with the severity of these symptoms and the treatment administered for *H. pylori*. Blood samples of 5–8 ml were collected from patients, with serum segments isolated and preserved in sterile Eppendorf tubes for immediate usage in both rapid *H. pylori* antibody testing and serum *H. pylori* IgG antibody detection via an enzyme immunoassay (ELISA) test. Furthermore, stool specimens were promptly collected from all subjects for the prompt identification of *H. pylori* antigens in stool. Additionally, the body mass index (BMI) was calculated by dividing the individual's weight by (height)^2^.

### Statistical analysis

A Statistical Package of Social Sciences (SPSS version 21) analyzed the data. The results presented as percentages, means, standard deviations (SD). In addition, *Chi*-square *X*^*2*^ test was used for categorical variables. Furthermore, comparison was made by independent sample t-test and one-way ANOVA. *P* values < 0.05 were considered as statistically significant.

### Ethical approval

The protocol of this work is approved by the Ethics Committee of Biological Sciences Department, Faculty of Science, Sana’a University, Yemen. This Ethics Committee agreed with the Helsinki Declaration for the Protection of Human Subjects. Informed consent was obtained from all the study participants.

## Results

### Association of socio-demographic characteristics of study subjects with confirmatory *H. pylori* tests

Initially, upon examining the demographic profiles of 200 patients diagnosed with *H. pylori* infections and their correlation with confirmatory diagnostic procedures, a noteworthy rise in the average age of the patients was observed. Out of all patients, a majority of 108 individuals (54%) were female, while 92 (46%) were male. Additionally, 148 patients (74%) reported the consumption of Khat (*Catha edulis*), 48 (24%) were identified as smokers, 127 (63%) had a familial history of *H. pylori* infections, and 60 (30%) had familial occurrences of PUD. Notably, the results of the SAT revealed a significant statistical association at (P < 0.05) with various factors including body mass index (BMI), intake of fruits and vegetables, recurrent infections, treatment protocols, repeated infections, and endoscopic assessments. Conversely, no significant correlations were detected with age, gender, residency, Khat consumption, smoking habits, familial predisposition to *H. pylori* infections and PUD. Moreover, a statistically significant correlation at (P < 0.05) was established between positivity to serum *H. pylori* IgG antibodies and parameters such as age, BMI, smoking history, familial incidence of *H. pylori* infections and PUD, recurrent infections, and treatment regimens. However, no significant association were observed concerning place of residence, gender, dietary habits regarding fruits and vegetables, or coffee consumption, as shown in Table 1.

**Table 1:** Association of Socio-demographic characteristics of study participants with confirmatory *H. pylori* tests.

	Variable	*SAT*		*P* value	*H. pylori IgG*		*P *value
		Positive (108)	Negative (92)		Positive (157)	Negative (43)	
Age (years)	Mean ± SD	39.41 ± 16.36	33.73 ± 14.22	0.744	24.37 ± 9.32	21.90 ± 8.74	0.001*
BMI (kg/m^2^)	Mean ± SD	33.75 ± 4.24	23.95 ± 4.47	0.015*	39.57 ± 6.46	26.69 ± 5.21	< 0.001*

	No. (%)	No. (%)	No. (%)		No. (%)	No. (%)	
Gender							
Male	92 (46)	52 (48.1%)	40 (43.5)	0.509	88 (56.1%)	21 (48.8%)	0.095
Female	108 (54)	56 (51.9%)	52 (56.5%)		69 (43.9%)	22 (51.2%)	
Residency							
Rural	110 (55)	63 (58.3%)	47 (51.1%)	0.305	68 (43.3%)	24 (55.8%)	0.145
Urban	90 (45)	45 (41.7%)	46 (48.9%)		89 (56.7%)	19 (44.2%)	
Education level							
Elementary	105 (52.5)	62 (57.4%)	43 (46.7%)	0.089	87 (55.4%)	18 (41.9%)	0.239
High school	54 (27)	30 (27.8%)	24 (26.1%)		41 (261%)	13 (30.2%)	
Professional	41 (20.5)	16 (14.8%)	25 (27.2%)		29 (18.5%)	12 (27.9%)	
Vegetables and fruit eating							
Yes	172 (86)	98 (90.7%)	74 (80.4%)	0.036*	131 (83.4%)	41 (95.3%)	0.046*
No	28 (14)	10 (9.3%)	18 (19.6%)		26 (16.6%)	2 (4.7%)	
*Khat (Catha edulis)* Chewing							
Yes	148 (74)	81 (75%)	67 (72.8%)	0.727	121 (77.1%)	27 (62.8%)	0.059
No	52 (26)	27 (25%)	25 (27.2%)		36 (22.9%)	16 (37.2%)	
Smoking							
Yes	48 (24)	27 (25)	21 (22.8%)	0.720	45 (28.7%)	3 (7%)	0.003*
No	152 (76)	81 (75)	71 (77.2%)		112 (71.3%)	40 (93%)	
Family history of *H. pylori*							
Yes	127 (63.5)	68 (63%)	59 (64.1%)	0.864	107 (68.2%)	20 (46.5%)	0.009*
No	73 (36.5)	40 (37%)	33 (35.9%)		50 (31.8%)	23 (53.5%)	
Family history of PUD							
Yes	60 (30)	33 (30.6%)	27 (29.3%)	0.853	54 (34.4%)	6 (14%)	0.010*
No	140 (70)	75 (69.4%)	65 (70.7%)		103 (65.6%)	37 (86%)	
Treatment							
Yes	109 (54.5)	51 (47.2%)	58 (63%)	0.025*	96 (61.1%)	13 (30.2%)	< 0.001*
No	91 (45.5)	57 (52.8%)	34 (37%)		61 (38.9%)	30 (69.8%)	
Recurrent infection							
Yes	89 (44.5)	58 (53.7%)	31 (33.7%)	0.005*	83 (52.9%)	6 (14%)	< 0.001*
No	111 (55.5)	50 (46.3%)	61 (66.3%)		74 (47.1%)	37 (86%)	
Endoscopy							
Yes	18 (9%)	14 (13%)	4 (4.3%)	0.034*	16 (10.2%)	2 (4.7%)	0.261
No	182 (91%)	94 (87%)	88 (95.7%)		141 (89.8%)	41 (95.3%)	

The results represented by frequency and percent %, *H. pylori*: *Helicobacter pylori*, *PUD* peptic ulcer disease, *BMI* body mass index, P ≤ 0.05 considered as significant.

### Prevalence of ***H. pylori*** infection according to different tests

The findings of the current study indicated that the prevalence of *H. pylori* infections as detected by rapid anti-*H. pylori* test encompassed all 200 patients (100%), while 157 individuals (78.5%) yielded positive results for serum *H. pylori* IgG antibodies, and 108 patients (54%) exhibited positive outcomes with the SAT methodology, as shown in Table 2.

**Table 2:** Prevalence of *H. pylori* infection according to different tests.

Variable	Number	Percent%
Rapid anti *H. pylori*		
Yes	200	100
No	0	0
Total	200	100
*H. pylori* IgG		
Yes	157	78.5
No	43	21.5
Total	200	100.0
SAT		
Yes	108	54.0
No	92	46.0
Total	200	100.0

The results represented by frequency and percent %, *SAT* Stool antigen test.

### Classification of different stages of ***H. pylori*** infections

**Figure 1:**
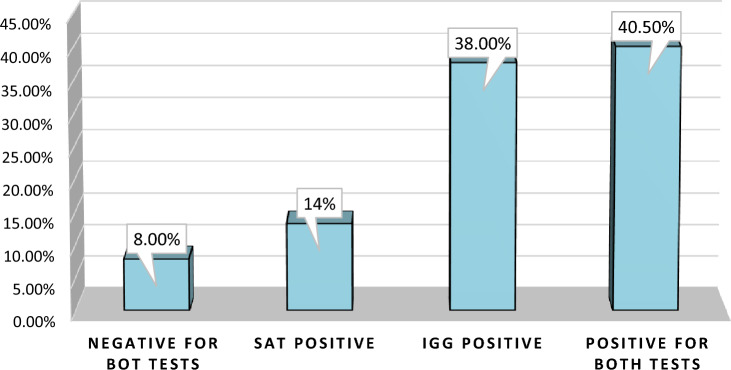
Classification of different stages of *H. pylori* infections.

Clinically, the findings of the current study demonstrated the categorization of distinct stages of *H. pylori* infections as follows: (1) a total of 16 patients (8%) exhibited negative outcomes in both the serum *H. pylori* IgG antibody and SAT confirmatory tests (indicative of absence of infections). (2) Furthermore, 27 individuals (13.5%) only tested positive in the SAT examination (reflecting acute infections). (3) 76 patients (38%) exclusively demonstrated positive results in the serum *H. pylori* IgG antibody test (signifying chronic infections). (4) Notably, 81 subjects (40.5%) were positive in both the serum *H. pylori* IgG antibody and SAT tests (indicative of active chronic infections), as shown in Fig. 1.

### The diagnostic value of serum ***H. pylori*** IgG antibody and stool antigen test

**Figure 2:**
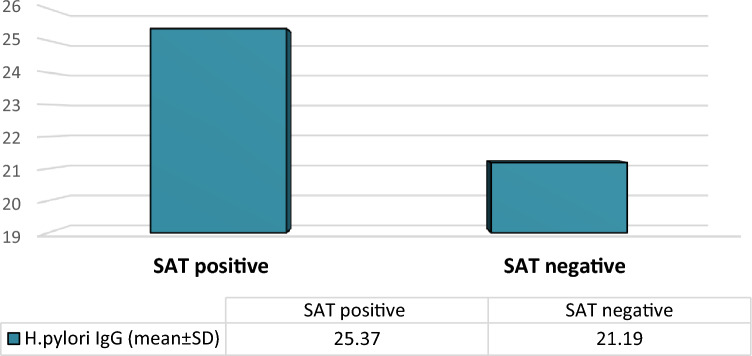
The diagnostic value of serum *H. pylori* IgG antibody and stool antigen test.

Significantly, the study's findings indicated that the diagnostic value of SAT and serum *H. pylori* IgG antibody for the diagnosis of *H. pylori* infections exhibited notably higher values in the SAT-positive groups (25.37 ± 10.47 NTU/ml) compared to those in the SAT-negative groups (21.193 ± 8.22), indicating a statistically significant difference (P < 0.05), as depicted in Fig. 2.

### Association between serum ***H. pylori*** IgG and SAT tests of ***H. pylori*** patients with gastrointestinal symptoms

Remarkably, the study revealed a significant correlation between SAT outcomes and gastrointestinal symptoms (inclusive of nausea, vomiting, heartburn, and abdominal pain), manifested by an increase in SAT positivity among symptomatic patients, as well as a significantly higher serum level of *H. pylori* IgG among symptomatic individuals. Additionally, the study indicated a linkage between *H. pylori* infection and vomiting, as evidenced by higher serum *H. pylori* IgG levels among patients experiencing vomiting as well as positivity to SAT (P < 0.05), as shown in Table 3.

**Table 3:** Association between serum *H. pylori* IgG and SAT tests of *H. pylori* patients with gastrointestinal symptoms.

Variable	*H. pylori* IgG	*P* value	SAT		X^2^	*P* value
	Mean ± SD		Positive No. (%)	Negative No. (%)		
Nausea						
Yes (138)	25.10 ± 9.53	˂ 0.001*	89 (82.4%)	49 (53.3%)	19.73	˂ 0.001*
No (62)	19.76 ± 8.59		19 (17.6%)	43 (46.7%)		
Vomiting						
Yes (99)	25.52 ± 10.51	0.002*	71 (65.7%)	28 (30.4%)	24.77	˂ 0.001*
No (101)	21.41 ± 8.06		37 (30.4%)	64 (69.6%)		
Heartburn						
Yes (116)	26.01 ± 9.73	˂ 0.001*	70 (64.8%)	46 (50%)	5.47	0.034*
No (84)	19.90 ± 8.11		38 (35.2%)	46 (50%)		
Abdominal pain						
Yes (150)	25.02 ± 9.85	˂ 0.001*	89 (82.4%)	61 (66.3%)	8.87	0.009*
No (50)	18.73 ± 6.75		19 (17.6%)	31 (33.7%)		

The results represented by frequency and percent %, Independent t test, *X*^*2*^: *Chi* square test *P ≤ 0.05 considered as significant.

### Association between serum ***H. pylori*** IgG and SAT tests with severity of gastrointestinal symptoms

Clinically, the study's results indicated a statistically significant association between SAT outcomes and the severity of gastrointestinal symptoms, with a heightened rate of SAT positivity observed with increased symptom severity. Moreover, the mean level of serum *H. pylori* IgG antibodies was significantly higher in the group exhibiting very severe symptoms (P < 0.05), as shown in Table 4.

**Table 4:** Association between serum *H. pylori* IgG and SAT tests with severity of gastrointestinal symptoms.

Symptoms	*SAT*		*P* value	*H. pylori IgG (mean* ± *SD)*	*P *value
	Positive (108) No. (%)	Negative (92) No. (%)			
Without symptoms	0	19 (20.7)	< 0.001*	14.97 ± 4.14	< 0.001*
Mild symptoms (1 symptom)	14 (13%)	16 (17.4%)		19.22 ± 6.37	
Moderate symptoms (2 symptoms)	21 (19.4%)	19 (20.7%)		22.79 ± 7.83	
Severe symptoms (3 symptoms)	37 (34.3%)	22 (23.9%)		25.73 ± 9.61	
Very Severe symptoms (4 symptoms)	36 (33.3%)	16 (17.4%)		26.89 ± 10.92	

The results represented by frequency and percent %, *X*^*2*^: *Chi* square test, Independent t test, *P ≤ 0.05 considered as significant.

### Association of serum ***H. pylori*** IgG and SAT tests with ***H. pylori*** infection duration

Lastly, it was observed that the serum *H. pylori* IgG levels were significantly elevated (P < 0.05) among patients with an infection duration exceeding 3 years. On the other hand, the association between the SAT status of study participants and the duration of *H. pylori* infection indicated no substantial variance in SAT test outcomes when compared against the duration of *H. pylori* infection (P > 0.05), as shown in Table 5.

**Table 5:** Association between serum *H. pylori* IgG and SAT tests with *H. pylori* infection duration.

Symptoms	*SAT*		*P* value	*H. pylori IgG (mean* ± *SD)*	*P *value
	Positive (108) No. (%)	Negative (92) No. (%)			
1 month ago	34 (31.5%)	17 (18.5%)	0.155	17.55 ± 8.36	< 0.001*
6 months ago	19 (17.6%)	22 (23.9%)		23.70 ± 8.04	
1–3 years	34 (31.5%)	29 (31.5%)		24.43 ± 8.38	
More than 3 years	21 (19.4%)	24 (26.1%)		28.51 ± 10.37	

The results represented by frequency and percent %, *X*^*2*^: *Chi* square test, independent t test, *P ≤ 0.05 considered as significant.

## Discussion

*Helicobacter pylori* infection remains a significant global public health concern, with an estimated 4.4 billion individuals worldwide testing positive for *H. pylori*. Chronic *H. pylori* infections are commonly acquired during childhood (4).

The study results revealed a significant association between SAT outcomes and BMI, consumption of vegetables and fruits, recurrent infections, treatments, as well as endoscopic procedures. Conversely, no significant correlations were found with age, gender, place of residence, consumption of Khat (*Catha edulis*), smoking habits, familial history of *H. pylori* infections, or PUD. Notably, there was a significant correlation between serum *H. pylori* IgG antibody levels and age, BMI, smoking habits, familial history of *H. pylori* infections and PUD, recurrent infections, and treatments. These results are consistent with previous studies^18,19^ that indicated the elderly population over 59 years to be most affected by *H. pylori* infections. A study conducted in Yemen^20^ revealed a peak SAT positivity of 60% in the age range of 40–80 years and 45.6% in the age group of 19–40 years. Similarly, a study from Saudi Arabia^21^ reported a higher prevalence of *H. pylori* infections using SAT in individuals over 50 years of age.

Regarding gender, the present study did not find any significant association between gender and seropositivity to *H. pylori* detected by serum *H. pylori* IgG antibody and SAT (P > 0.05), contrasting findings from another study^18^ that reported a statistically significant link between *H. pylori* seropositivity and gender. The current study aligns with study by^22,23^ which also found no significant gender-based differences in *H. pylori* infection. Furthermore, there was no significant disparity in *H. pylori* SAT outcomes based on gender (P > 0.05).

Consistent with a study by^24^, the findings of this study demonstrated a higher *H. pylori* positivity rate in patients who chewed Khat (*Catha edulis*). However, another study^25^ contradicted these results, suggesting no significant difference in *H. pylori* infection rates based on Khat chewing (*Catha edulis*). Divergent outcomes have been documented in various studies regarding the correlation between *H. pylori* infection and smoking habits.

In a case–control study conducted in Nairobi, a higher prevalence of *H. pylori* infection was observed among among Khat chewers (*Catha edulis*), indicating a potential link between Khat (*Catha edulis*) consumption and susceptibility to gastrointestinal disorders^26^. Another study also suggested a greater occurrence of gastrointestinal disorders among Khat (*Catha edulis*) chewers, implying that Khat (*Catha edulis*) consumption might predispose individuals to gastrointestinal issues beyond just *H. pylori* infection^27^. Additionally, a study indicated a significant association between Khat (*Catha edulis*) chewing and duodenal ulcers, potentially attributed to stress related to Khat (*Catha edulis*) consumption^28^ Furthermore, a separate study found a higher prevalence of *H. pylori* infection among individuals who engaged in Khat (*Catha edulis*) chewing^29^.

Various previous studies have highlighted smoking as a risk factor for different diseases, including gastric cancer and *H. pylori* infection^25,30,31^. The current study's findings revealed a significantly elevated presence of *H. pylori* IgG antibodies in smokers compared to non-smokers, with a statistically significant distinction. These findings contrasted with those from^32^, which indicated no significant difference in *H. pylori* infection rates between smokers and non-smokers (P > 0.05).

Concerning residency, the results of the current study aligned with those of^33^, indicating no significant difference in *H. pylori* infection rates between patients residing in urban and rural areas (P > 0.05).

Significantly, the study's findings highlighted a statistically significant variance in serum levels of *H. pylori* IgG antibodies and SAT concerning BMI (P < 0.05). These findings were in line with findings of many previous studies^34–38^.

The mechanisms of association between *H. pylori* and obesity are varied, involving gastrointestinal hormones such as ghrelin and leptin, pivotal in metabolic regulation and energy balance. Ghrelin, produced in the stomach, stimulates food intake, while leptin functions in the opposite direction. Studies have reported lower serum levels of leptin and ghrelin in patients with *H. pylori* infections. Moreover, insulin resistance, a significant risk factor for several metabolic disorders including obesity, may be influenced by *H. pylori* infection. Obesity's interaction with *H. pylori* infection is being explored, with indications that the immune milieu in obese individuals may support *H. pylori* survival^39,40^.

In relation to a family history of PUD, the present study's results concurred with those from^41^, showing an association between *H. pylori* infection and a PUD history (P < 0.05). However, these findings contradicted those of^42^, which indicated no statistically significant difference in *H. pylori* infection positivity based on a family history of PUD. The potential association of *H. pylori* infection with a family history of the infection might be attributed to inter-familial transmission, emphasizing close and intimate contact among family members as a crucial mode of *H. pylori* spread^43^.

In this study, the consumption of vegetables and fruits showed a significant association with *H. pylori* infection. This could be attributed to the unhealthy conditions in Yemen, inadequate access to clean water sources, and insufficient awareness regarding the necessity of thoroughly washing fruits and vegetables. Previous studies have suggested that fruits and vegetables could be exposed to contamination through irrigation with polluted water. Moreover, the main sources for *H. pylori* include soil, water, animal waste, and human feces^44–46^.

Furthermore, the study findings indicated that the prevalence of *H. pylori* infections detected through rapid tests stood at 100%, while serum *H. pylori* IgG positivity was at 78.5%, and the rate of *H. pylori* infection positivity using SAT among the participants was 54%. These results were consistent with a study of^47^, which reported IgG seropositivity for *H. pylori* in 48.3% of the study subjects and a SAT positivity rate of 28.2%. A study conducted in Yemen^20^, showed a blood antibody test positivity of 72% and SAT positivity of 49%. Other studies^19,48^ indicated higher serum antibody positivity compared to stool antigen positivity. However^49^ found a SAT positivity rate of 82%, while^50^ revealed a lower SAT positivity rate of 32.22% among participants. The detection of *H. pylori* stool antigen and UBT are reliable indicators of an active infection^51^. On the other hand, serological tests for *H. pylori* IgG antibodies denote a past or chronic infection, with a decrease in *H. pylori* shedding observed with the progression of chronic infections, a pattern often seen in elderly individuals^52^.

The current study revealed distinct stages of *H. pylori* infections: 16 (8%) of the patients were negative for both serum *H. pylori* IgG antibody and SAT (no infections), 27 (13.5%) were only SAT positive (acute infections), 76 (38%) were only serum *H. pylori* IgG positive for both the serum *H. pylori* IgG antibody and SAT (chronic infections), and 81 (40.5%) were positive for both (active chronic infections). A study conducted in Ethiopia reported similar findings^53^.

Importantly, the study demonstrated that SAT and serum *H. pylori* IgG antibody had higher diagnostic value in SAT-positive groups (25.37 ± 10.47 NTU/ml) compared to SAT-negative groups (21.193 ± 8.22) at (P < 0.05). This aligns with previous studies^54,55^, reporting a seroprevalence of 78.4% using *H. pylori* serum IgG ELISA. However, the findings contrasted with a study^56^ from KSA showing a lower prevalence of 28.3% using ELISA for *H. pylori* IgG detection.

The results of this study also indicated a significant association between SAT and gastrointestinal symptoms like nausea, vomiting, heartburn, and abdominal pain. Patients with symptoms had higher SAT positivity and serum *H. pylori* IgG levels. The study also showed that vomiting was particularly linked to *H. pylori* infection, with higher IgG levels among patients experiencing vomiting and positive SAT results. Similar associations were found in previous studies linking epigastric pain, heartburn, and vomiting to *H. pylori* infection^57–60^.

Moreover, the findings of the current study demonstrated a significant correlation between SAT positivity and the severity of gastrointestinal symptoms, with a higher rate of SAT positivity observed in individuals with more severe symptoms. Additionally, there was a statistically significant increase in the mean level of serum *H. pylori* IgG antibody among participants experiencing very severe symptoms. Another study explored the link between *H. pylori* infection and symptom severity, noting that moderate to severe symptoms were associated with RUT positivity compared to individuals with mild symptoms^61^.

Finally, patients with an *H. pylori* infection duration exceeding 3 years exhibited significantly elevated levels of serum *H. pylori* IgG. On the other hand, no significant difference was observed in the association between the SAT status of participants and the duration of *H. pylori* infection when compared. The prevalence of active chronic *H. pylori* infections was notably high, primarily attributed to key risk factors such as advancing age, smoking, Khat consumption (*Catha edulis*), family history of *H. pylori* infection, presence and severity of gastrointestinal symptoms, and prolonged duration of *H. pylori* infection.

## Data Availability

The data are not publicly available due to privacy or ethical consideration. The data sets generated during and/or analyzed during the current study are available from the corresponding author upon request.
